# Capillary orbits

**DOI:** 10.1038/s41467-019-11850-1

**Published:** 2019-09-02

**Authors:** Anaïs Gauthier, Devaraj van der Meer, Jacco H. Snoeijer, Guillaume Lajoinie

**Affiliations:** Physics of Fluids Group and Max Plank Center Twente. Mesa + Institute and Faculty of Science and Technology, J.M. Burgers Centre for Fluid Dynamics and Max Plank Center Twente for Complex Fluid Dynamics, University of Twente, P.O. Box 217, 7500 AE Enschede, The Netherlands

**Keywords:** Self-assembly, Wetting, Fluid dynamics

## Abstract

Millimeter-sized objects trapped at a liquid surface distort the interface by their weight, which in turn attracts them towards each other. This ubiquitous phenomenon, colloquially called the “Cheerios effect” is seen in the clumping of cereals in a breakfast bowl, and turns out to be a highly promising route towards controlled self-assembly of colloidal particles at the water surface. Here, we study capillary attraction between levitating droplets, maintained in an inverse Leidenfrost state above liquid nitrogen. We reveal that the drops spontaneously orbit around each other – mirroring a miniature celestial system. In this unique situation of negligible friction, the trajectories are solely shaped by the Cheerios-interaction potential, which we obtain directly from the droplet’s dynamics. Our findings offer an original perspective on contactless and contamination-free droplet cryopreservation processing, where the Leidenfrost effect and capillarity would be used in synergy to vitrify and transport biological samples.

## Abstract (web summary)

It is known that liquid drops can be levitated by continuous vapour flow above a liquid surface. Here the authors combine the ultra-low friction provided by the bath with the interaction force between two drops due to interface deformations to study the dynamics of interactions between multiple objects.

## Introduction

The Cheerios effect is an everyday-life phenomenon that can be seen in the clumping of cereals in a bowl or that of bubbles at the surface of a sparkling liquid. The origin of the interaction between floating objects lies in the distortion of the liquid surface^1–3^, which typically generates attractive forces^4–8^. As such, the Cheerios effect offers a simple strategy for self-assembly of colloids^9,10^, controlled by tuning the shape^11^ or wetting properties of the particles^12^. Capillary interactions also play a crucial role in the life of water walking creatures and pond vegetation^4,5^, and are used to reach food, escape predators^13^ and disperse seeds^14^. The interaction between two particles placed on a liquid-air interface depends on a subtle equilibrium of gravity, buoyancy, and surface tension. In the limit of small interfacial deformations, however, the attractive potential energy is simply expressed as the product of the weight of one floating particle by the surface deformation caused by the second one^3^. In other words, each particle warps the space around it (the bath surface) proportionally to its mass and evolves in the gravitational potential landscape generated by its counterparts. Thus, the floating bodies form an intriguing microcosm that is reminiscent of general relativity. Such a comparison has been suggested before, and the long-range interaction between colloids has been proposed as a way to mimic the gravitational collapse of galaxies^15^. The substantial drag experienced by floating objects, however, fundamentally distinguishes them from their celestial counterparts.

Here, in by-passing the drag we push the comparison further: we show that levitating particles submitted to capillary attraction follow a variety of intricate orbits. The trajectories, shaped by the Cheerios interaction potential fundamentally differ from the usual Newtonian conics. Making use of the absence of friction, we directly derive the Cheerios interaction potential from the particle dynamics, and model the experimental trajectories. We finally discuss the possibility of obtaining bounded orbits.

## Results

### Orbiting trajectories of frictionless droplets

**Fig. 1:**
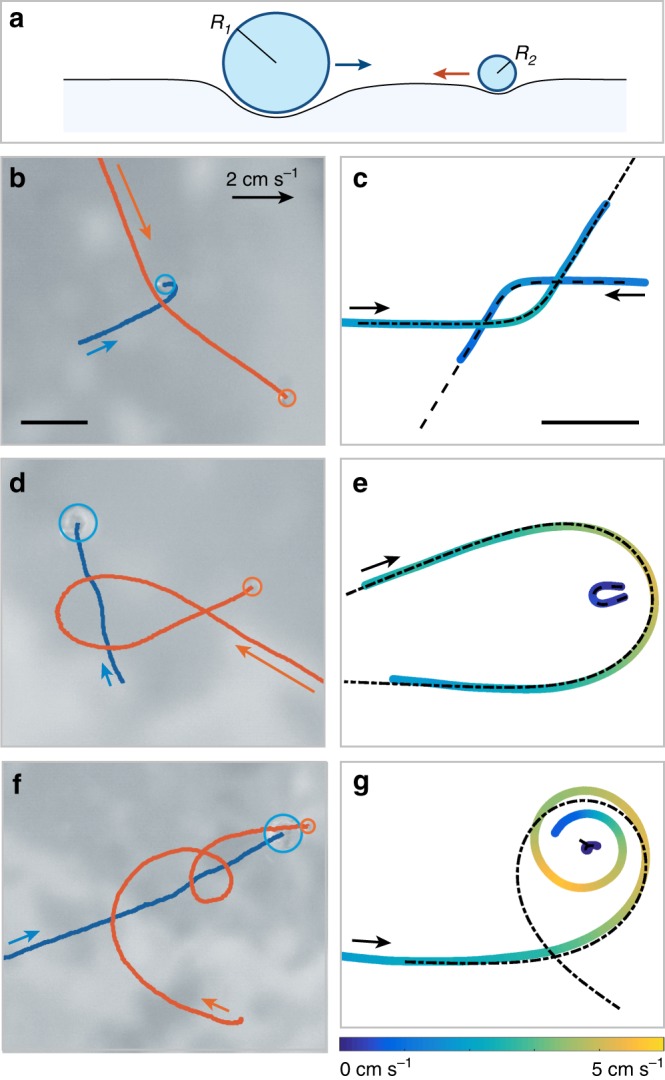
Orbiting trajectories of two cryogenic skaters. **a** Two silicone oil drops with radii *R*_1_ and *R*_2_, deposited on a liquid nitrogen bath, are maintained in an inverse Leidenfrost state and spontaneously self-propel. We study their dynamics as they approach each other, in conditions of negligible friction. **b** Deflection between two drops (*R*_1_ = 410 μm, in blue and *R*_2_ = 360 μm, in orange), as seen experimentally. See also Supplementary Movie 1. The arrows indicate the initial direction of motion and velocity of the particles, and the scale bar corresponds to 3 mm. **c** Same experiment as in (**b**), now plotted in the center of mass frame. The scale bar indicates 3 mm. **d** Deviation of a small drop (*R*_2_ = 390 μm) when approaching a bigger drop (*R*_1_ = 810 μm). See also Supplementary Movie 2. **e** Drop trajectories of (**d**) in the center of mass frame. **f** Collision trajectory between two frozen marbles (*R*_1_ = 820 μm, *R*_2_ = 260 μm). See also Supplementary Movie 3. **g** Drop trajectories of (**f**) in the center of mass frame. The scale is the same in panels **b**, **d**, and **f**, and panels **c**, **e**, and **g**. In panels **c**, **e**, and **g** the color code indicates the drop velocity, from 0 cm s^−1^ (dark blue) to 5 cm s^−1^ (yellow). The dotted lines show the corresponding theoretical trajectories calculated from the capillary potential *E*(*r*)

We observe the motion of two silicone oil drops (with radii *R*_1_ and *R*_2_ between 250 μm and 1.4 mm) gently released above a quiescent bath of liquid nitrogen. As illustrated in Fig. 1a, each drop is kept in an “inverse Leidenfrost” state above the liquid surface, a levitating state that is enabled by the continuous vapor flow produced by the cryogenic bath^16–19^. A unique feature of this system is that, in the absence of any physical contact with the bath, friction forces remain extremely small^20–22^, and the rapid motion resembles usual Leidenfrost drops that are highly mobile when placed into external fields^23^. In addition, in our experiment, this small drag is almost perfectly compensated by a small propulsion force caused by a symmetry breaking within the film sustaining the drops^19^. The drops thus behave like nearly frictionless “cryogenic skaters”: they glide in perfectly straight trajectories and, once frozen, they keep a constant velocity *v*_0_ ranging from 1 to 3 cm s^−1^. Levitation and propulsion are maintained as the drops cool down, generating frozen marbles subjected to capillary interactions only.

Figure 1b, d, f provides top-view images of close encounters of such skaters, illustrating the trajectories of each particle. The arrows indicate the direction and magnitude of the initial velocities. On the right, the panels c, e, and g represent the same trajectories as in b, d, and f, respectively, plotted in the center of mass frame. The color code indicates the drop velocity which typically varies between 0 cm s^−1^ (blue) and 5 cm s^−1^ (yellow). Figure 1b provides an example of a small deflection between two particles of similar size (Supplementary Movie 1). The fast particle (orange) is deflected by 35°, while the slow particle (blue) almost performs a U-turn. The trajectory becomes more intuitive in the reference frame of the center of mass (Fig. 1c), with a deflection angle (measured relatively to the incoming direction) of 58 ± 2° for both droplets. At first glance, the trajectories resemble a miniature version of the hyperbolas of classical gravitational orbits. However, deflections often exceed the maximum value possible for open orbits in Newtonian gravity, namely 180°, as in Fig. 1d. In the center of mass frame (Fig. 1e), the small particle is indeed deflected by 220 ± 3°. In this case the drop masses differ by a factor 10 and only the smaller skater is deviated (Supplementary Movie 2). More generally, deflection angles can exceed 360°, and the drops do more than a complete revolution before escaping. The highest deflection observed is close to 3 full revolutions, although most of the particles that experience a deviation exceeding 270° eventually collide. Figure 1f provides an example of such a collision for a mass ratio of 30 (Supplementary Movie 3). Here as well, only the smallest particle is substantially deflected. It follows the other in a spiral trajectory and eventually collides with it after more than a turn. Depending on the droplets velocity and impact angle, a collision can eventually lead to a capture (as in Fig. 1g), but not always. A durable contact can happen after multiple small rebounds (Supplementary Movie 4), but collisions can also lead to rebounds large enough for the drops to eventually escape their mutual attraction (Supplementary Movie 5). In addition, “gravitational assist” events (Supplementary Movie 6), were observed where a small drop is accelerated as it passes near a large drop.

### Interaction potential

Interestingly, the absence of friction in our experiment offers a direct measure of the capillary interaction potential *E*. In the Nicolson approximation where the deformations are small, the interaction potential between two nonwetting objects (with a contact angle of 180°) at a distance *r* and with masses *m*_1_ and *m*_2_ reads^3^

1 $$E(r) = m_1m_2\frac{{\left( {\rho g} \right)^2}}{{2\pi \gamma \rho _N^2}}K_0\left( {r{\mathrm{/}}a} \right) = Cm_1m_2K_0\left( {r{\mathrm{/}}a} \right),$$

which can also be viewed as an interaction between point particles^24^. In this expression *g* stands for gravity, and *γ*, *ρ*, and *ρ*_*N*_ are the surface tension and the densities of the particle and the bath, respectively. The function $K_0(r{\mathrm{/}}a)$ is the zeroth-order modified Bessel function of the second kind, and reflects the distortion of the interface induced by *m*_1_, which is felt by *m*_2_ (and vice versa). The lengthscale $a = \sqrt {\gamma /\rho _Ng}$ is the capillary length of the bath. At distances $r \,< \,a$, the function $K_0(r/a)$ diverges logarithmically in analogy with two-dimensional Newtonian gravity and electrostatics. For $r \,> \,a$, however, the interactions decay exponentially. To experimentally verify this formula, we consider a system of two drops with masses *m*_1_ and *m*_2_ moving toward each other. We reformulate the two-body system into a one-body problem with a reduced mass $m_r = \frac{{m_1m_2}}{{m_1 + m_2}}$. In the absence of dissipation, the capillary potential *E*(*r*) can then be directly inferred from the kinetic energy *E*_*k*_ of the reduced particle, using $E(r) = E_k - E_k\left( {r = \infty } \right)$.

**Fig. 2:**
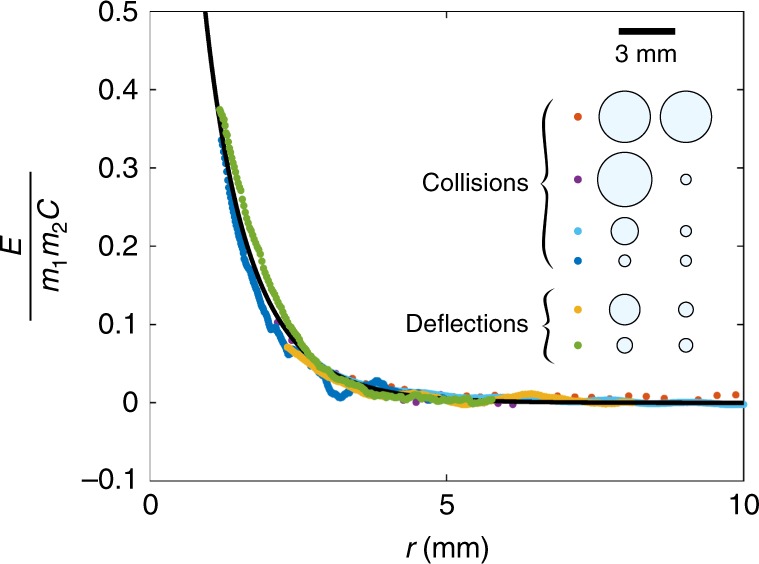
Experimental measurement of the Cheerios potential. The experimental (nondimensional) potential $E(r)/(Cm_1m_2)$ is extracted from the dynamics of a pair of particles. Each color corresponds to a different experiment, with particles radii and mass ratio systematically varied. Experimental data are compared with the theoretical shape of the potential $K_0(r/a)$, plotted as a black line

In Fig. 2, we selected four head-on collisions and two small deviations, and plotted the dimensionless capillary potential $E(r)/(m_1m_2C)$, as inferred from the particle dynamics. Each experiment is represented in a different color, as indicated in the inset. The particle radii are systematically varied between 290 μm and 1.4 mm. All experimental data collapse onto a single curve, in excellent agreement with the theoretical prediction $K_0(r/a)$, without any adjustable parameters, demonstrating that the far-field expression of the interaction potential (1) still holds when the droplets are close enough to collide. The small deformation criterion is indeed valid even for the largest drops, that can only interact at distances *r* > *R*_1_ + *R*_2_. At these relatively large distances, the bath deformation is always smaller than 100 μm.

### Capillary orbits

With the expression for *E*(*r*) in hand, we can use Eq. (1) to compute particle trajectories such as reported in Fig. 1. Like gravity, the potential is proportional to the product of the two masses, but depends on particle distance in a different manner. Here, we systematically explore how the well-known celestial orbits are affected upon replacing the 1/*r*-potential by this unusual, Bessel-shaped form $K_0(r/a)$.

As a test, we first numerically integrate the equations of motion, using the initial conditions corresponding to the experiments in Fig. 1. The initial velocity (taken within values compatible with the noise in the experimental data) is chosen to obtain the best fit, by root mean square error minimization. The obtained trajectories are superimposed as dotted lines and compared directly to the experiments. The resulting predictions provide an almost perfect fit for the experimental trajectories in Fig. 1c, e. However, it fails to reproduce the dynamics in Fig. 1g: the calculated trajectory predicts a large deflection without collision. More generally, it turns out that the model accurately describes the majority of our experiments for small deflections and collisions, while it systematically underestimates the amplitude of the deflection when the particles are deflected by more than 270°. As we will discuss below, we attribute this to a small but nonnegligible loss of energy by friction, occurring when the drops experience substantial changes in velocity.

A systematic classification of the capillary orbits (without friction) is obtained following the standard approach for central force fields^25^. We introduce the effective capillary potential *U*_eff_ of the associated particle with reduced mass *m*_*r*_

2 $$U_{{\mathrm{eff}}} = \frac{{L^2}}{{2m_rr^2}} + E(r),$$

accounting for the orbit’s angular momentum $L = m_rr^2\dot \theta$.

**Fig. 3:**
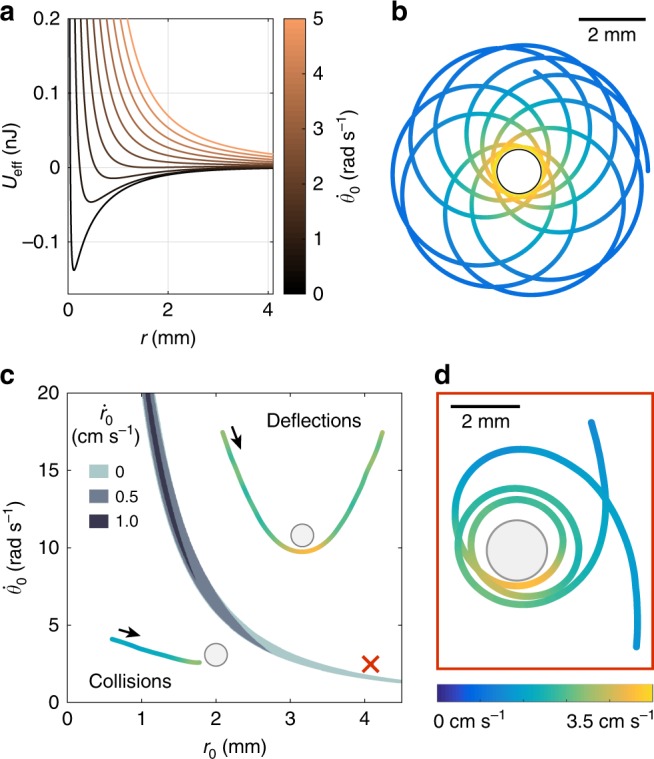
Classification of capillary orbits. **a** Effective potential *U*_eff_ as a function of distance *r*, for varying initial angular velocity $\dot \theta _0$. The particle masses and their initial distance are kept constant ($m_r = 9.10^{ - 8}$ kg, and *r*_0_ = 4.1 mm). **b** Example of a bounded orbit for this potential, with initial conditions $\dot \theta _0$ = 1.465 rad s^−1^ and $\dot r_0$ = 0. See also Supplementary Movie 7. **c** Phase diagram to classify orbits, expressed using the initial conditions *r*_0_, $\dot \theta _0$ for $m_r = 9.10^{ - 8}$ kg. The upper region corresponds to deflections and the lower region to collisions, both illustrated by experimental trajectories. Bounded orbits without collisions are only observed in a narrow band plotted in shades of gray, for varying radial velocities $\dot r_0$. The initial conditions of (**d**) are indicated by a red cross. **d** Experimental trajectory close to a bounded orbit in the center of mass frame. See also Supplementary Movie 8

Figure 3a shows *U*_eff_ with varying *L*, for typical experimental conditions (see caption). In contrast to scale-free algebraic potentials, *U*_eff_ exhibits a minimum only for sufficiently small *L*, which for the chosen parameters (reduced mass $m_r = 9.10^{ - 8}$ kg, and initial distance *r*_0_ = 4.1 mm) corresponds to an initial angular velocity $\dot \theta _0 \,<\, 1.6$ rad s^−1^. This restriction is a direct consequence of the screening of the interaction beyond the capillary length *a*. Bounded orbits are possible when a minimum is present, an example of which is given in Fig. 3b (and Supplementary Movie 7). The bounded states exhibit flower-like patterns rather than closed trajectories which are, according to Bertrand’s theorem^26,27^, a special feature of the −1/*r* and *r*^2^ potentials. A prime example of this effect is given by the precession of mercury’s perihelion, due to relativistic corrections to the 1/*r* potential. In the example of Fig. 3b, the precession angle is equal to 109°.

We now provide in Fig. 3c a classification of orbits for typical initial conditions. Figure 3c is roughly divided into two regions, illustrated by experimental trajectories. The upper part corresponds to deflections, for which *U*_eff_ does not have a minimum. In the lower quadrant, bounded orbits are theoretically possible: however, the trajectories cross the “collision radius” *R*_1_ + *R*_2_, and the particles come into contact. Bounded trajectories that stay outside the collision radius appear only in a very narrow window illustrated in shades of gray (for thee different initial radial velocities $\dot r_0$). As $\dot r_0$ increases, the thin gray area shrinks even further and eventually disappears (for $\dot r_0 \,> \, 1.5$ cm s^−1^), which illustrates the extreme experimental difficulty in attaining bounded capillary orbits. We could nonetheless observe trajectories in the vicinity of the bounded orbits region, an example of which is given in Fig. 3d (Supplementary Movie 8). Initial conditions are indicated by a red cross in the phase diagram. The angular momentum and radial velocity are just a little too high to allow for a bounded trajectory, and the particle indeed undergoes an impressive deflection, close to three full rotations (1065 ± 3°), but eventually escapes the attractive field.

## Discussion

**Fig. 4:**
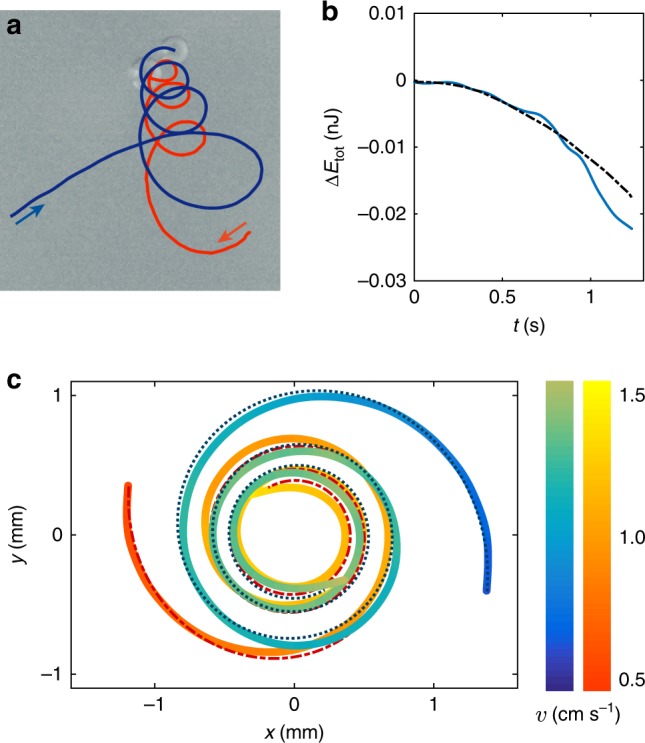
The effect of energy loss. **a** Experimental trajectory of two particles with radii *R*_1_ = 360 μm and *R*_2_ = 340 μm. See also Supplementary Movie 9. **b** Energy loss $\Delta E_{{\mathrm{tot}}}$ of a two-drop system as a function of time (in blue). The modeled Δ*E* is plotted with a dotted line. **c** Trajectories of the two particles in the center of mass frame, with two different color codes for their velocities. The dotted lines are the modeled trajectories, calculated numerically by integrating (for each particle) a small friction force with magnitude $F = \frac{{\eta _vR^2}}{h}\left( {V - v_0} \right)$ and a direction opposite to the particle velocity *V*

Though friction is in itself small, the accumulated loss of energy during strong deflections can ultimately turn a deflection into a collision. An example of this is given in Fig. 4 and Supplementary Movie 9 for a case at the upper limit of the region of bounded orbits. The two frozen drops (highlighted in blue and orange in Fig. 4a) eventually collide after a long spiral lasting two and a half turns. The total energy $E_{{\mathrm{tot}}}$, which is constant when the particles are far, decreases slowly but steadily prior to collision (Fig. 4b). We interpret this as the effect of an additional friction, occurring as the particles accelerate. Indeed, before approaching each other, the self-propelled drops glide at their terminal velocity *v*_0_, for which propulsion and friction cancel out. They thus behave as perfectly frictionless objects and keep a constant energy. However, once accelerated (at a velocity $V \,> \, v_0$) by the attractive potential, each particle experiences an effective friction force *F* arising from the increased hydrodynamic drag within the supporting vapor film. In the laboratory frame, this effective drag force has a magnitude $F = \alpha \frac{{\eta _vR^2}}{h}\left( {V - v_0} \right)$ and a direction opposite to the particle velocity *V*. Here *η*_*v*_ is the viscosity of nitrogen vapor, *h* the thickness of the vapor film, and *α* a numerical prefactor. In Fig. 4, the drops are frozen to liquid nitrogen temperature, and *h* is fixed by the spontaneous evaporation of the bath, which maintains them in levitation. Following^19^, *h* is calculated: it is close to 10 μm for the two (almost identical) drops considered here. Integrating the equations of motion with this friction force provides a very good fit to the spiraling trajectory of Fig. 4c, with a prefactor *α* equal to 1. The color code (in blue and orange) indicates the velocity of each drop, and the dotted lines are the best numerical fit. A similar agreement is also obtained when fitting the collision trajectory of Fig. 1g (see Supplementary Fig. 1). In addition, Fig. 4b shows that our model also nicely reproduces the loss of energy in time. Small friction forces, as discussed here, arise as the drop accelerates but only become significant for large deviations. This is why, upon selecting simple trajectories such as in Fig. 2, one can accurately measure the capillary potential without any hindrance from drag.

Our results unveil the capacity of frictionless particles to act as sensitive probes, which can be used for the direct measurement of the forces that govern capillary self-assembly. Control over capillarity as demonstrated in the current study is of paramount importance for rising applications in, e.g., drop cryopreservation^28–30^: cryogenic levitation provides a unique method for rapid droplet vitrification^16,18^ with minimal contamination hazards, while at the same time offering a highly versatile procedure to remotely select, manipulate and organize such biological samples for optimal handling and conservation.

## Methods

### Experimental procedure

Silicone oil drops (with a density *ρ* = 930 kg m^−^^3^ and viscosity *η* = 9.3 mPa s) are release a few centimeters above the surface of a still liquid nitrogen bath. Large drops (with radius *R* > 750 μm) are generated using calibrated Hamilton needles, while smaller ones (250 μm < *R* < 750 μm) are obtained using stretched glass capillaries. The smallest drops (*R* < 500 μm) are generated by transferring the liquid attached to a larger capillary on a thinner one, from which it slides and fall. The liquid nitrogen bath is contained in a small beaker with characteristic size 5 cm. To avoid boiling, the small beaker is placed on a copper disk at the center of a sacrificial bath of liquid nitrogen^17,19^. The sacrificial bath has a diameter of 19 cm and is filled with 5 cm of liquid nitrogen. The boiling of the sacrificial bath generates a nitrogen atmosphere and partially insulates the central bath, which keeps a still surface. Both beakers are placed in a homemade polystyrene cryostat, with internal size 20 × 20 × 15 cm^3^ and 4 cm thick walls. Experiments are filmed from the top, at typically 500 fps using a high-speed camera (Photron Mini UX-100). The box cover is removed for each experiment (that typically lasts 1 min), and then put back to ensure insulation. Drop trajectories are finally tracked using a home-made Python algorithm.

## Supplementary information

Supplementary Information

Description of Additional Supplementary Files

Supplementary Movie 1

Supplementary Movie 2

Supplementary Movie 3

Supplementary Movie 4

Supplementary Movie 5

Supplementary Movie 6

Supplementary Movie 7

Supplementary Movie 8

Supplementary Movie 9

## Data Availability

The data that supports this study is available upon request from the corresponding author.
