# Supplementary material for: Capillary orbits
Source: Nat Commun. 2019 Sep 2;10:3947. doi: 10.1038/s41467-019-11850-1 (PMC6718406; doi:10.1038/s41467-019-11850-1)
Supplement: Supplementary file 1 — Supplementary Information [file 41467_2019_11850_MOESM1_ESM.pdf]

Capillary orbits
Supplementary informations

A. Gauthier *et al.*

Supplementary figures

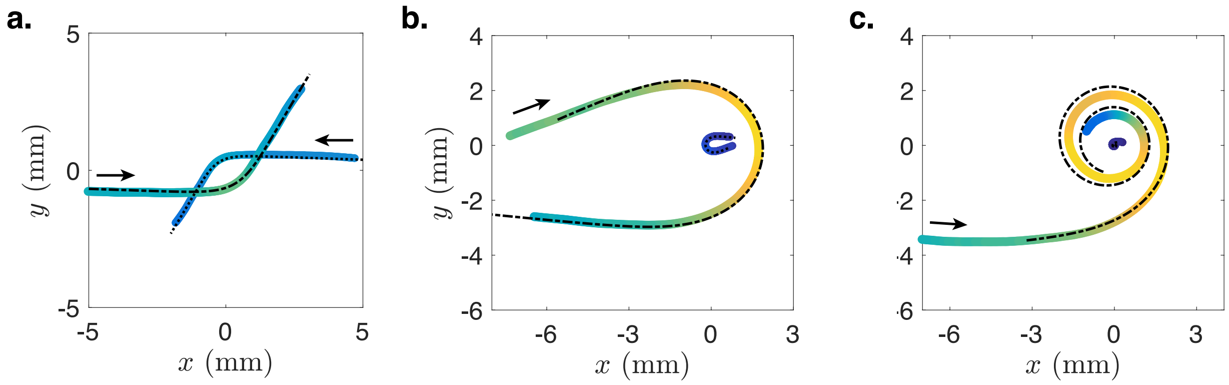

Supplementary Figure 1: **Orbiting trajectories fitted with friction.** **a**. Deflection between two drops ($R_1 = 410$ µm and $R_2 = 360$ µm), as seen in the center of mass frame. The arrows indicate the initial direction of motion of the particles. **c**. Deviation of a small drop ($R_2 = 390$ µm) when approaching a bigger drop ($R_1 = 810$ µm). **c**. Collision trajectory between two frozen marbles ($R_1 = 820$ µm, $R_2 = 260$ µm). The color code indicates the drop velocity, from 0 cm/s (dark blue) to 5 cm/s (yellow). The dotted lines are the theoretical trajectories, calculated numerically by integrating a small friction force F $= \frac{\eta_v R^2}{h}(V - v_0)$ for each particle.
