# Supplementary material for: Capillary orbits
Source: Nat Commun. 2019 Sep 2;10:3947. doi: 10.1038/s41467-019-11850-1 (PMC6718406; doi:10.1038/s41467-019-11850-1)
Supplement: Supplementary file 2 — Description of Additional Supplementary Files [file 41467_2019_11850_MOESM2_ESM.docx]

**Description of Additional Supplementary Files**

**File Name: Supplementary Movie 1**

**Description:** Deviation between two small drops, with the trajectories marked. The particles have a radius *R_1_* = 410 μm (in blue) and *R_2_* = 360 μm (in orange). The movie is slowed down 10 times.

**File Name: Supplementary Movie 2**

**Description:** Large deflection of a small drop (with radius *R_2_* = 390 μm) by a larger drop (*R_1_* = 810 μm). The deflected angle is 220 ± 3 degrees, beyond the Newtonian maximum of 180 degrees. The movie is slowed down 10 times.

**File Name: Supplementary Movie 3**

**Description:** Collision between a small drop (*R_2_* = 260 μm) and a large drop (*R_1_* = 820 μm). The movie is slowed down 10 times.

**File Name: Supplementary Movie 4**

**Description:** Collision between two drops with identical radius *R* = 350 μm. After 6 damped rebounds, the two frozen drops stay attached to each other. The movie is slowed down 10 times.

**File Name: Supplementary Movie 5**

**Description:** Collision between two drops (*R_1_* = 390 μm, *R_2_* = 370 μm), resulting in a rebound: the two drops eventually escape their mutual attractive field. The movie is slowed down 10 times.

**File name: Supplementary Movie 6**

**Description:** Gravity assist of a drop with radius (*R_2_* = 360 μm) when passing by a larger drop (with radius *R_1_* = 760 µm).

**File name: Supplementary Movie 7**

**Description:** Example of a theoretical bounded orbit for a particle with reduced mass $m_{r}$ = 9.10^−8^ kg. The initial position and velocity of the corresponding reduced particle are $r_{0}$ = 4.1 mm, $\dot{\theta_{0}}$= 1.465 rad s^-1^, $\dot{r}_{0}$ = 0 cm s^-1^.

**File name: Supplementary Movie 8**

**Description:** Experimental trajectory the closest to a bounded orbit, obtained for two particles with radii *R_1_* = 450 μm and *R_2_* = 320 μm. In the frame of reference of the reduced particle, the initial conditions are $m_{r}$ = 9.10^−8^ kg, $r_{0}$ = 4.1 mm, $\dot{\theta_{0}}$ = 2.5 rad s^-1^, $\dot{r}_{0}$= 0.7 cm s^-1^. The latter two values are just a little too high to enable a closed orbit: the total deflection reaches 6π before the two particles move apart from each other. The movie is slowed down 10 times.

**File name: Supplementary Movie 9**

**Description:** Experimental spiraling collision with a large deflection of two and a half turns. The energy decreases slowly but progressively as the two drops spiral around each other, which turns a bounded orbit into a collision. The drop sizes are *R_1_* = 360 μm and *R_2_* = 340 μm, and the movie is slowed down 10 times.
